# Changes in leisure time physical activity, obesity-related morbidities, fatal and non-fatal CVD events and total mortality: Over 20-year follow-up in the opera study

**DOI:** 10.1371/journal.pone.0342429

**Published:** 2026-02-23

**Authors:** Asla Suutari-Jääskö, Karri Parkkila, Hannu Vähänikkilä, Mikko Tulppo, Juha Perkiömäki, Heikki Huikuri, Olavi Ukkola

**Affiliations:** 1 Research Unit of Biomedicine and Internal Medicine, Medical Research Center Oulu, Oulu University Hospital, University of Oulu, Oulu, Finland; 2 Northern Finland Birth Cohorts, Arctic Biobank, Infrastructure for Population Studies, Faculty of Medicine, University of Oulu, Oulu, Finland; Universiti Malaya, MALAYSIA

## Abstract

**Background:**

The impact of changes in leisure time physical activity (LTPA) is not well-documented, especially when considering occupational physical activity (OPA). This study examines the effects of LTPA changes in workers with varying physical activity demands.

**Methods:**

Part of the OPERA study, we tracked morbidities for over 20 years (P1, from 1993 to 2014) and mortality for over 8 years (P2, from 2014 to 2021–2022) with 599 participants. They were categorized into four LTPA groups (“sedentary,” “started,” “quit,” “active”) and two OPA groups (“office workers” and “occupationally physically active”).

**Results:**

Maintaining regular LTPA was associated with lower incidence of hypertension, diabetes and metabolic syndrome (p-values 0.007, < 0.001 and <0.001 respectively). Non-fatal cardiovascular disease (CVD) events were more common (p = 0.006, HR 1.99, CI95% 1.22–3.26) in the “quit” group during P1, especially among “occupationally physically active” (p < 0.001, HR 2.29, CI95% 1.23–4.29). During P2, fatal CVD events were associated with being in the “sedentary” group (p = 0.042, HR 2.67, CI95% 1.04–7.03). This association was particularly evident among “office workers,” where belonging to the “sedentary” and “quit” groups was associated with a higher risk of fatal CVD events (p = 0.017, HR 5.45, CI95%1.36–21.91, and p = 0.025, HR 4.55, CI95% 1.21–17.19, respectively). Furthermore, total mortality was associated with being in the “sedentary” or “quit” groups (p = 0.029, HR 3.69, CI95% 1.14–11.93, and p = 0.009, HR 4.61, CI95%1.47–14.49, respectively).

**Conclusions:**

Stopping LTPA in middle age was associated to higher risk for non-fatal CVD events in “occupationally physically active” individuals. Fatal CVD events were associated with a sedentary lifestyle in whole study population. Among “office workers,” both a sedentary lifestyle and stopping regular LTPA were associated with higher risks of fatal CVD events and all-cause mortality.

## Introduction

The benefits of physical activity have been widely known for a long time in the previous literature. More recently, the literature has observed that the benefits of physical activity are only related to leisure time physical activity. The phenomenon is called the physical activity paradox. [1, 2] Previous studies have noted that especially high OPA can be harmful since it could increase the risk of overall and CVD mortalities. [3] However, it has been observed that when socioeconomic status is taken into account, moderate to high OPA could even prolong the life expectancy. [4] A previously conducted systematic review studied the benefits of LTPA in different OPA study subjects. The study reported that LTPA was beneficial in lowering mortality among all study subjects, but the benefits from LTPA were smaller in workers with high OPA. [5] In our previous study, we noticed that high OPA study subjects benefited more from lower amounts of LTPA and higher amounts of LTPA were harmful for high OPA workers. [6] Altogether, the knowledge of the association between LTPA and OPA is still widely unknown in the literature. There are fundamental differences between LTPA and OPA. LTPA is often more controlled in terms of intensity, frequency, duration and recovery. The intensity and duration of OPA is too low or too long and it is performed without sufficient recovery time to improve cardiorespiratory health. [2, 3] The current PA recommendations do not take into consideration the source of physical activity [7, 8].

When it comes to overall mortality and CVD mortality, previous studies have noted that maintaining or increasing LTPA has been observed to be beneficial. [9, 10] Increasing LTPA from any baseline has been found to be beneficial for CVD mortality. [11] A previous study with a short follow-up also noted that there were no significant differences in CVD mortality between study subjects who were regularly active and those who started to exercise regularly. [12] Only few studies have brought up the association of LTPA and OPA. These studies reported high sedentary behavior at work and overall to be associated with increased mortality. Reducing the amount of sitting time at work or increasing LTPA could eliminate the risk related to sedentary behavior [9, 13].

Our primary objective was to determine whether changes in LTPA over 20 years affect cardiovascular disease incidence differently in “office workers” versus occupationally physically active workers. The secondary objectives were to investigate the association between changes in LTPA and all-cause mortality across occupational groups, to assess the relationship between LTPA changes and the incidence of type 2 diabetes and hypertension, and to identify optimal patterns of LTPA for individuals in different occupational categories.

## Subjects and methods

The OPERA study (Oulu Project Elucidating the Risk of Atherosclerosis) is a population-based epidemiological investigation aimed at identifying risk factors and clinical outcomes related to atherosclerotic cardiovascular diseases. In its first phase, OPERA was conducted as a cross-sectional study involving middle-aged individuals. The initial study cohort comprised 600 individuals with hypertension (aged 40–59 years, 50% men) and 600 age- and sex-matched non-hypertensive controls, all residing in the city of Oulu. The hypertensive participants were identified from the register of the Social Insurance Institution of Finland (Kela) as those entitled to a special reimbursement for hypertension medication granted after August 1980. To ensure balanced age distribution, participants were randomly selected using age-stratified sampling, with 15 men and 15 women chosen for each birth year from 1931 to 1950. For each hypertensive subject, a corresponding control subject—matched by age and sex but not entitled to special reimbursement for hypertension treatment—was randomly selected from the national health register. Individuals managing mild hypertension without medication were included in the control group, resulting in a population that also captured undiagnosed and untreated hypertension cases. Study invitations and reminders were mailed by Kela, with the recruitment period spanning from December 10, 1990, to September 15, 1992. In total, 1,045 from 1,200 invited individuals participated (519 with hypertension and 526 without), yielding a participation rate of 87.1%. Clinical data became available for research on August 15, 1994.

In the second phase (2013–2014) the study subjects were recruited for a follow-up visit. The current study focuses on the follow-up study of the OPERA study. In 2013–2014, surviving members of the cohort (aged 63–83 years) were invited to a study visit at the Department of Internal Medicine. The Social Insurance Institution of Finland (Kela) sent invitation letters to 813 individuals (213 had died earlier, 2 could not be reached (abroad), 17 died during the mailing period). The main reason for not participating in the follow-up study was the patient’s poor condition and/or illness. 48 lived far away and could not participate. The participation (n 600) was 73.8% (281 men – 319 women; 290 hypertensive – 310 controls) (14). In the present study the total number of study subjects was 599, since one study subject was rejected because of unemployment at the baseline.

Clinical examinations included weight, height, waist and hip measurements and blood pressure measurements. Body mass index (BMI) was calculated as weight (kg) divided by height squared (m^2^). A questionnaire presented to all participants provided information on smoking habits, alcohol consumption, physical activity (LTPA and OPA), use of medication, and past medical history, including diseases. Alcohol consumption was calculated as grams of absolute alcohol consumed per week and smoking as the number of cigarettes smoked per day. The lifetime smoking burden was calculated as pack-years (1 pack-year = 20 cigarettes smoked/day in one year). [14] As definition of metabolic syndrome we used the IDF (International Diabetes Federation) criteria. Hypertension was defined as a blood pressure (BP) > 140/90 mmHg or current antihypertensive medication. Type 2 diabetes was determined according to the WHO criteria. [15] A wide range of routine laboratory analyses were conducted.

### Laboratory analyses

The laboratory tests were carried out in Joint Municipal Service Provider of Northern Finland Laboratory Center, NordLab Oulu (after 12 h fasting) using Siemens Advia 1800 chemistry and Siemens Advia Centaur XP immunochemistry analyzers (Siemens Healthcare Diagnostics Oy). After fasting blood had been drawn, the subjects were given a 75 g glucose load. Both 1 h and 2 h glucose and insulin concentrations were determined, except for previously known insulin-treated diabetics. The glucose concentrations were measured with the glucose dehydrogenase method (Diagnostica, Merck, Darmstadt, Germany). The serum insulin levels were measured using a two-site immunoenzymometric assay (AIA-PACK IRI, Tosoh corp., Tokyo, Japan). The concentrations of total cholesterol and triglycerides in the plasma and lipoprotein fractions were determined by enzymatic colourimetric methods (kits of Boehringer Diagnostica, Mannheim GmbH, Germany, catalogue nos. 236691 and 701912, respectively), using a Kone Specific, Selective Chemistry Analyser (Kone Instruments). Very low-density lipoproteins (VLDL) were isolated by ultracentrifugation, and high-density lipoproteins (HDL) were separated from the VLDL-free fraction using heparin–manganese precipitation. Low-density lipoprotein (LDL) cholesterol was calculated by subtracting HDL cholesterol from the cholesterol content of the VLDL-free fraction [16].

### Outcome classification

Morbidities, non-fatal CVD events and general measurements were obtained during P1 follow-up period (P1) from 1991 to 2014. Total mortality was studied in the follow-up period (P2) from 2014 to 2022. Information on total mortality was obtained from the Finnish Causes of Death Register after the year 2022, (8-year follow-up, P2). Data on the occurrence of non-fatal CVD events were collected from the Hospital Discharge Register after 2014. [17] A non-fatal CVD event was defined as the first occurrence of either a major coronary heart disease (CHD) event or a stroke, excluding subarachnoid hemorrhage (SAH). CHD was identified based on the presence of ICD-10 codes I20.0, I21, or I22, or ICD-8/9 codes 410 or 4110 as the primary diagnosis, or I21 and I22 (ICD-10)/ 410 (ICD-8/9) listed as secondary or tertiary diagnoses. Additionally, a history of coronary artery bypass grafting or angioplasty qualified as a CHD event. CHD-related mortality was determined using ICD-10 codes I20–I25, I46, R96, and R98, or ICD-8/9 codes 410–414 and 798 (excluding 7980A), recorded as underlying or contributing causes of death. Stroke, excluding SAH, was identified using ICD-10 codes I61, I63 (excluding I636), and I64; ICD-9 codes 431, 4330A, 4331A, 4339A, 4340A, 4341A, 4349A, and 436; and ICD-8 codes 431 (excluding 43101 and 43191), 433, 434, and 436, based on their presence as primary, secondary, or tertiary diagnoses or causes of death. [6] Major adverse cardiovascular events (MACE) included ischemic stroke, TIA, coronary heart disease events (unstable angina, non–ST-elevation myocardial infarction, ST-elevation myocardial infarction), and already diagnosed coronary heart disease.

### Determination of LTPA and OPA classes

The study subjects were originally divided into four LTPA groups by modifying a scale developed by Saltin and Grimby. [18] The first group, (no exercise), included individuals who were sedentary aside from light housework. The second group (irregular), engaged in occasional activities such as walking or cycling. The third group, (regular) exercised regularly 1–2 times per week for over 30 minutes per session. The fourth group, (heavy regular), activity group exercised more than three times per week for over 30 minutes, typically involving activities like jogging, swimming, or cycling. We limited the leisure time physical activity at the baseline to two, regularly active and sedentary. Regularly active subjects (regular and heavy regular) exercised more than 1–2 times per week for more than 30 min. Sedentary (no exercise and irregular) study subjects did not meet this criterion. After the P1 the changes in LTPA status were determined and subjects were thus divided into four groups. The first group, “sedentary”, was formed by those who were sedentary both at the baseline and after P1. Subjects in the “started”, were sedentary at the baseline but started exercising regularly during the P1. The third group, “quit”, exercised regularly at the baseline but quit during the follow-up. Subjects in the “active” exercised regularly throughout the study. We analyzed morbidities and mortality among the whole population, but we also considered study subjects’ OPA background. The baseline OPA represented the burden of OPA, since most subjects retired during P1. The classification of the OPA groups is described in a previous study. [6] Due to the small number of study subjects in the physically demanding occupations we limited the OPA groups into two, “office workers” and “occupationally physically active”. The “office workers” reported in the baseline questionnaire that they were in a sedentary occupation with little or no movement during the working day. The “occupationally physically active”, were moderately to highly physically active during the working day at the baseline. [19] Educational level was assessed at follow-up and used as an indicator of lifetime educational attainment. “Low” educational level corresponded to primary education (primary education), “medium” to vocational or upper secondary education (secondary education), and “high” to university degree or equivalent (tertiary education). Educational level was included as a socioeconomic factor in the Cox regression analyses of CVD events, CVD mortality, and all-cause mortality.

### Statistical methods

Data were analyzed using IBM SPSS Statistics version 29. The chi-squared test (χ2) was used for categorical variables. Statistical significance was set at p < 0.05. Analysis of variance (ANOVA) was used to compare more than two groups with normally distributed continuous variables. Normal and skewed distributions were determined graphically. Post-hoc analyses of differences between groups were conducted using Tukey’s statistical test. The overall effect size was determined using ANOVA, from which eta squared was obtained. The effect size of between-group differences was further assessed with an Independent T-test, from which Cohen’s d was calculated. Clinical impact was evaluated using thresholds of 0.20 (small), 0.50 (medium), and 0.80 (large). The statistical clinical impact was compared against MCID values in accordance with best practices, when available, to assess the final clinical impact. For weight change, an MCID of greater than 5% change in body weight was considered significant, and for waist circumference, a change of more than 5 cm was considered significant. Skewed continuous variables (alcohol and smoking) were analyzed using the Kruskal–Wallis test with pairwise comparisons adjusted by the Bonferroni method. Effect sizes were examined with the Mann–Whitney U test, and clinical significance was assessed using Cliff’s delta and the rank-biserial correlation (r_rb). The thresholds applied were 0.147 for small, 0.33 for medium, and 0.474 for large effects.

Kaplan Meier survival curves were created using the R Core Team (2024). R: A Language and Environment for Statistical Computing. R Foundation for Statistical Computing, Vienna, Austria. < https://www.R-project.org/ > . Total mortality, fatal and non-fatal cardiovascular events were assessed as the cumulative proportional probability of development of deaths and CVD events and analyzed using Kaplan-Meier survival curves for physical activity groups. Statistical significance of the Kaplan-Meier survival curves was calculated using the log-rank test. The secondary survival analysis considering confounding factors was performed with Cox proportional hazards models. The proportional hazards assumption was assessed graphically.

### Ethical considerations

This study was approved by the Ethics Committee of the Medical Department of the University of Oulu (48/2009). Written informed consent for the use of their clinical records was obtained from all participants.

## Results

**Fig 1:**
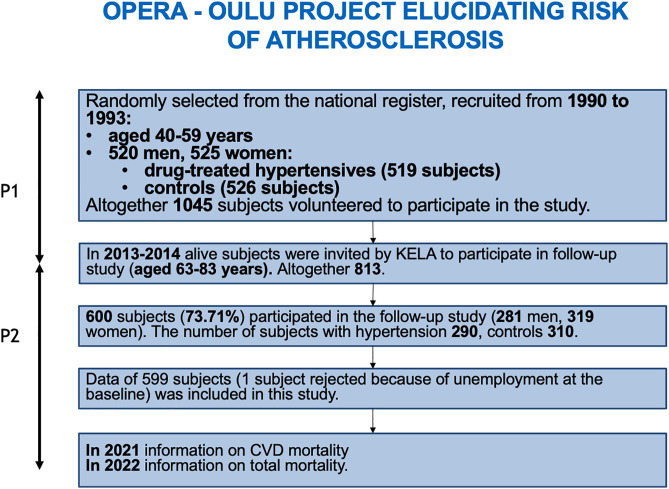
Flowchart of the study design, OPERA – Oulu Project Elucidating Risk of Atherosclerosis.

Of the original study population of 1,045 individuals, 813 were alive and residing in Finland at the end of the follow-up period (Fig 1). Of these, 600 participated in the second phase of the study, of whom 599 were eligible for inclusion. Thus, the overall participation rate was 73.7%. Those who took part were two years younger than the dropouts, their BMI, waist circumference, LDL-cholesterol levels were lower compared with the non-attendees. They consumed alcohol and smoked less and were more often female. LTPA did not differ. However, they were more often “office workers”. Those who took part had less artery disease and diabetes.

### Baseline characteristics (Table 1)

In the “quit” group, females were underrepresented (p = 0.039). At baseline, the “active” group had the lowest BMI, weight, and waist circumference (p < 0.001). The effect size for these variables was moderate, and the clinical impact was significant. The “active” group also smoked the least (p < 0.001). At baseline, the pack-years, was lowest in the “active” group compared with the other groups. Nevertheless, the clinical significance of this difference was considered small. There were no statistically significant differences in educational level between the LTPA groups (p = 0.065). Overall, participants with higher education constituted the smallest subgroup, with 86 individuals (14%). A total of 247 participants (41%) had completed vocational or institute-level education, and 266 participants (45%) had only basic education. The corresponding proportions for each LTPA group are presented in Table 1.

**Table 1:** Clinical characteristics during P1 follow-up.

Variable	1 Sedentary (N = 97)	2 Started (N = 103)	3 Quit (N = 87)	4 Active (N = 312)	P-value	Post-hoc	Effect size	Clinical impact
**Baseline Demographics**								
Age, years	49.6	49.5	51.1	50.1	0.219		0.273	Small
Female, %	54.6	48.5	41.4	57.7	0.039			
**Baseline Anthropometrics**								
Height, cm	167	169	170	168	0.266		0.202	Small
Weight, kg	81.5	81.0	82.0	74.4	<0.001	c,f***,e**	0.531	Significant
BMI	29.0	28.3	28.5	26.3	<0.001	c,e,f***	0.633	Significant
Waist, cm	93.8	90.9	93.3	86.3	<0.001	c,f***,e**	0.605	Significant
**Lifestyle Factors**								
Smoking, pack years	11.2	9.3	10.6	4.8	<0.001	c***,f**,e*	0.270	Small
Alcohol, g/wk	57.3	59.4	61.5	44.8	0.404		0.083	Small
**Education %**								
Basic	49	38	56	42	0.065			
Vocational	42	42	35	43	0.065			
Higher	9	20	9	15	0.065			
**20-Year Changes**								
BMI	2.7	1.0	2.3	1.5	0.001	a**,c,d*	0.420	Significant
Weight, kg	4.3	−0.2	3.2	1.5	0.005	a**	0.384	Significant
Waist, cm	10.0	6.5	10.2	6.6	<0.001	c**,a,d,f*	0.375	Significant
Alcohol	−8.2	−22.1	−16.3	−9.5	0.310		0.047	Small
Smoking	4.6	3.2	4.0	2.1	0.017	c*	0.151	Small

Post hoc comparisons for continuous variables: a) 1 vs. 2; b) 1 vs. 3; c) 1 vs. 4; d) 2 vs. 3; e) 2 vs. 4; f) 3 vs. 4. Significance levels: *p < 0.05, **p < 0.01, ***p < 0.001. Effect size thresholds for continuous normally distributed variables were defined according to Cohen’s d: 0.20 (small), 0.50 (medium), and 0.80 (large). Clinical significance was further evaluated in relation to the MCID. Effect size thresholds for continuous skewed variables according to Cliff’s delta: 0.147 small, 0.33 medium, 0.474 significant.

During P1 follow-up, BMI increased the least in the “started” and “active” groups. The BMI change was significant when comparing the “started” group to the “sedentary” and “quit” groups and between the “active” and “sedentary” groups (p = 0.001). The greatest weight gain was observed in the sedentary group, whereas a slight reduction was noted in the started group (p = 0.005). Waist circumference increased the most in the “sedentary” and “quit” groups (p < 0.001), with significant differences between the “active” and “quit” groups mentioned above. These changes were on the borderline between small and medium in terms of statistical effect size. Nevertheless, considering the MCID values for weight gain and waist circumference, the clinical relevance was considerable.

### The frequency of morbidities (Table 2)

#### At baseline.

Those who exercised regularly (“active”) had the lowest incidence of HTN, while “sedentary” and “started” groups had the highest (p = 0.004). This trend was also seen in “occupational physically active” subjects (p = 0.032). The “sedentary” group had the highest frequency of metabolic syndrome (MetS), and the “active” group had the lowest (p < 0.001). Similar results were found both in “office workers” and “occupationally physically active”, when analysing OPA groups separately (p < 0.001–0.004). At baseline, the “quit” group had the highest number of MACE (p < 0.001). When analyzing OPA separately, a similar pattern was observed among occupationally physically active workers (p < 0.001), whereas no differences between groups were found among “office workers” (p = 0.078). At the baseline there were no difference in the incidence of diabetes (p = 0.272).

**Table 2:** Morbidities, whole study population during P1 follow-up.

Endpoint Group	Activity Group	Baseline Prevalence (%)	20-year Prevalence (%)	20-year Cumulative incidence (%)	20-year Incidence Rate,/1000 PY	Absolute Risk Reduction, %	Clinical Significance, NNT
**HTN**							
	Sedentary	59 (60,8)	81 (83,5)	22 (57,9)	29,0	2,4	42
	Started	61 (59,2)	81 (78,6)	20 (47,6)	23,8	7,6	14
	**Quit**	**44 (50,6)**	**71 (81,6)**	**27 (62,8)**	**31,4**		
	Active	136 (43,6)	216 (69,2)	80 (45,5)	22,7	8,7	12
***p-value***		*0.004*	*0.007*				
**DM**							
	**Sedentary**	**9 (9,3)**	**51 (52,6)**	**42 (47,7)**	**23,9**		
	Started	8 (7,8)	43 (41,7)	35 (36,8)	18,4	5,5	19
	Quit	8 (9,2)	35 (40,2)	27 (34,2)	17,1	6,8	15
	Active	15 (4,8)	86 (27,6)	71 (23,9)	12,0	11,9	9
***p-value***		*0.272*	*<0.001*				
**MetS**							
	**Sedentary**	**53 (54,6)**	**76 (78,4)**	**23 (52,3)**	**26,1**		
	Started	37 (35,9)	67 (65,0)	30 (45,5)	22,7	3,4	30
	Quit	37 (42,5)	62 (71,3)	25 (50,0)	25,0	1,1	91
	Active	68 (21,8)	166 (53,3)	98 (40,2)	20,8	5,3	19
***p-value***		*<0.001*	*<0.001*				
**MACE**							
	Sedentary	7 (7,2)	26 (26,8)	19 (21,1)	10,6	8,4	12
	Started	3 (2,9)	25 (24,3)	22 (22,0)	11,0	8,0	13
	**Quit**	**16 (18,4)**	**43 (49,4)**	**27 (38,0)**	**19,0**		
	Active	14 (4,5)	88 (28,2)	74 (24,8)	12,4	6,6	16
***p-value***		*<0.001*	*<0.001*				

The highlighted group in the table represents the reference group. HTN = hypertension, DM = diabetes mellitus, MetS = metabolic syndrome, MACE = major adverse cardiovascular event; includes acute myocardial infarction (AMI), stroke, and established coronary artery disease (CAD), PY = person years, NNT = number needed to treat.

#### At the end of the P1 follow-up.

At the end of P1 the “active” group still had the lowest incidence of HTN (p = 0.007). Absolute risk reduction compared to “quit” group was 8.7% and NNT 12. Among OPA groups, “office workers” had the lowest HTN incidence (p = 0.021), but not “occupationally physically active” (p = 0.258). The “active” group had the lowest incidence of diabetes, while the “sedentary” group had the highest (p < 0.001). Absolute risk reduction in the “active” was nearly 12% compared to “sedentary” and the NNT was 9. The “sedentary” and “office worker” groups developed diabetes most frequently (p = 0.002). Overall, it seemed that “occupationally physically active” individuals had fewer cases of diabetes with the “active” group having the lowest incidence (p = 0.021). The “active” group also experienced the least Mets, and the “started” group had lower MetS frequency than the “sedentary” and “quit” groups (p < 0.001), ARR being 5,3% and NNT 19 in the “active” compared to “sedentary”. Similar results were seen in OPA groups (0.007–0.008). The “quit” group had the highest incidence of MACE (p < 0.001). The “sedentary” and “started” groups showed the largest absolute risk reductions (8.4% and 8.0%, respectively), corresponding to NNT of 12 and 13 when compared with the “quit” group. The prominence of the “quit” group in terms of MACE was also evident when analyzing the two OPA groups separately (p = 0.023 for “office workers” and p = 0.028 for occupationally physically active participants).

After the P1 follow-up in 2014, no differences in disease prevalence were observed between the OPA groups. Likewise, within the LTPA change groups, no significant differences in disease prevalence were observed between “office workers” and “occupationally physically active” participants. Eventhough it seemed that fewer diabetes cases was found in “occupationally physically active”.

#### Evaluation of secondary prevention.

We explored differences in secondary prevention between OPA groups. In whole population at the baseline the “quit” group had the highest use of hypercholesterolemia (HC) drugs (5.7%, p = 0.043) and after P1 the “started” and “active” groups had the lowest use of HC drugs (p = 0.028). However, after the P1 follow-up among “occupationally physically active” subjects, a non-significant increase in HC drug use was observed in the “quit and “sedentary” groups (p = 0.055), as well as a borderline change in LDL levels in the “quit” group (p = 0.052). In the “office workers” there were no differences in the use of HC drug between LTPA groups (p = 0.312 and 0.55). Although, in the “office workers,” there was a significant difference in LDL change between the “quit” (dLDL −1.1) and “active” (dLDL −0.4) groups (p = 0.012). There were no significant differences in antiplatelet or anticoagulant use between “office workers” and “occupationally physically active” subjects.

### The occurrence of non-fatal CVD events (P1), fatal CVD events and mortality (P2)

**Fig 2:**
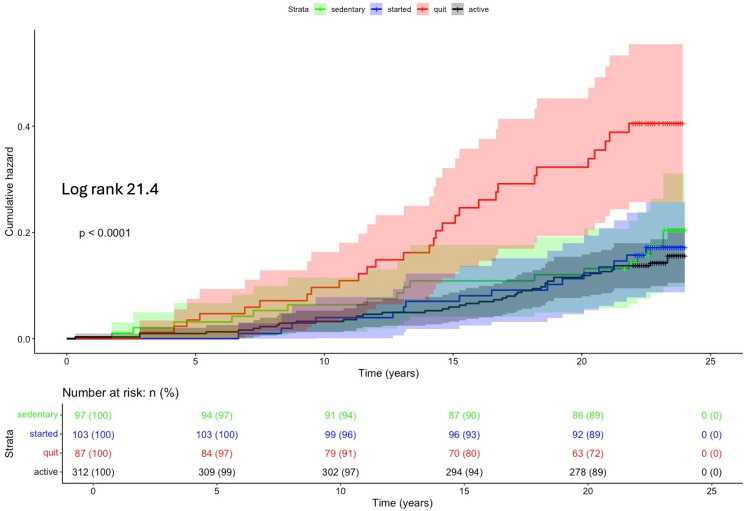
Kaplan-Meier cumulative hazard analysis for non-fatal cardiovascular events during 20-year P1 follow-up among leisure time physical activity change groups. Whole study population.

During the P1 follow-up, the “quit” group had the highest incidence of non-fatal CVD events (p < 0.001) (Fig 2). Compared to the “active” group, the hazard ratio (HR) for the “quit” group was 1.99 (p = 0.006, CI95% 1.22–3.26) (Table 3). In “occupationally physically active” subjects, but not among “office workers”, the “quit” group also had increased non-fatal CVD events (p < 0.001), with an HR of 2.44 (p = 0.005, CI95% 1.31–4.56). At baseline, no significant differences were observed between OPA groups when comparing non-fatal CVD events according to changes in LTPA. However, in the “quit” group there appeared to be a non-significant trend toward more frequent non-fatal CVD events among the occupationally physically active.

**Table 3:** Multivariable Cox regression analysis for non-fatal cardiovascular events during 20-year P1 follow-up (1993–2014) – whole study population (n = 599).

Variable	HR (95% CI)	p-value	Events, n (%)	Survival rate, %	NNH/ Clinical interpretention
**Leisure Time Physical Activity Pattern**					
Active (n = 312)	1 (reference)		42 (13.5)	86,5	–
Sedentary (n = 97)	1.12 (0.61–2.06)	0.707	16 (16.5)	83,5	33
Started (103)	1.14 (0.64–2.05)	0.652	16 (15.5%)	84,5	50
Quit (n = 87)	1.99 (1.22–3.26)	0.006	29 (33.3%)	66,7	5
**Demographic Characteristics**					
Sex (male)	2.27 (1.44–3.57)	<0.001			Higher risk compared to females
Age (years)	1.06 (1.02–1.10)	0.001			Risk increases with age
**Education**					
High	1				No significant association
Low	1.24 (0.65-2.35)	0.519			No significant association
Medium	0.85 (0.43-1.67)	0.636			No significant association
**Cardiovascular Risk Factors**					
Hypertension	1.39 (0.92–2.10)	0.116			No significant association
Diabetes	1.36 (0.72–2.60)	0.344			No significant association
LDL-cholesterol	1.21 (0.97–1.52)	0.098			No significant association
Smoking (pack-years)	1.00 (0.98–1.01)	0.629			No significant association
**Baseline Cardiovascular Disease**					
MACE baseline	3.06 (1.79–5.21)	<0.001			Strong predictor of non-fatal CVD events

During the P2 follow-up (2014–2021), an increased risk of CVD mortality was observed in the “sedentary” group in the whole study population (p = 0.012). Compared with the “active” group, the hazard ratio (HR) for the “sedentary” group was 2.67 (p = 0.042, 95% CI 1.04–7.03) (Table 4). When the OPA groups were analyzed separately, differences between the LTPA change groups were evident only among “office workers”, but not among the “occupationally physically active” (p = 0.003 and 0.462). Among “office workers”, both the “sedentary” and “quit” groups had an increased risk of mortality. Compared with the “active” group, the HR was 5.45 for the “sedentary” group (p = 0.017, 95% CI 1.36–21.91) and 4.55 for the “quit” group (p = 0.025, 95% CI 1.21–17.19). In the overall study population, survival was 89% and 90% in the “sedentary” and “quit” groups, and 95% and 97% in the “started” and “active” groups, respectively. Among “office workers”, the survival percentages were 87%, 82%, 96%, and 97%, while among “occupationally physically active” individuals, the corresponding survival rates were 92%, 96%, 94%, and 97%. When comparing fatal CVD events according to changes in LTPA, office workers in the “quit” group showed higher CVD mortality compared with occupationally physically active participants (p = 0.025; survival rates 82.4% vs. 96.2%). After adjustment, however, this difference was no longer statistically significant (p = 0.180, HR 4.13, CI95% 0.52–33.00).

**Table 4:** Multivariable Cox regression analysis for fatal cardiovascular events during P2 follow-up (2014–2021) – whole study population (n = 599).

Variable	HR (95% CI)	p-value	Events n (%)	Survival Rate(%)	NNH/ Clinical Interpretention
**Leisure Time Physical Activity Pattern**					
Active (n = 312)	1 (reference)		9 (2.9%)	97	–
Sedentary (n = 97)	2.67 (1.04–7.03)	0.042	10 (10.3%)	89	14
Started (103)	1.42 (0.47–4.29)	0.540	5 (4.9%)	95	50
Quit (n = 87)	1.90 (0.70–5.19)	0.211	8 (9.2%)	90	16
**Demographic Characteristics**					
Sex (male)	1.84 (0.84–4.02)	0.129			No significant association
Age (years)	1.11 (1.04–1.19)	0.002			Risk increases with age
**Education**					
High	1				No significant association
Low	0.35 (0.13-0.96)	0.041			Significant association
Medium	0.52 (0.19-1.44)	0.206			No significant association
**Cardiovascular Risk Factors At The Follow-Up**					
Hypertension	6.08 (0.80–46.30)	0.081			No significant association
Diabetes	1.79 (0.84–3.83)	0.133			No significant association
LDL-cholesterol	0.85 (0.55–1.32)	0.469			No significant association
Smoking (pack-years)	1.00 (0.99–1.02)	0.630			No significant association
**Follow-Up Cardiovascular Disease**					
MACE	1.75 (0.79–3.90)	0.169			No significant association

**Fig 3:**
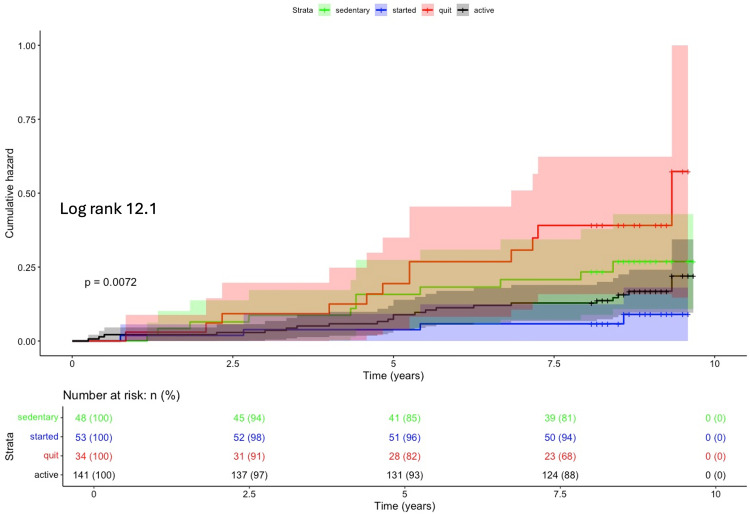
Kaplan–Meier cumulative hazard analysis for total mortality during 8-year P2 follow-up among leisure-time physical activity change groups, office workers.

During P2 (2014–2022), “office workers” showed a significant difference in short-term total mortality (p = 0.001, n = 276) (Fig 3). Compared to the “started” group, total mortality increased in the “quit” and “sedentary” groups (p = 0.009, HR 4.61 CI95% 1.47–14.49 and p = 0.029, HR 3.69; CI95% 1.14–11.93) (Table 5). Among occupationally active subjects or in the whole population, total mortality did not differ based on changes in LTPA habits. Regarding total mortality, no significant differences were observed between OPA groups in relation to changes in LTPA.

**Table 5:** Multivariable Cox regression analysis for total mortality during P2 follow-up (2014–2022) – office workers.

Variable	HR (95% CI)	p-value	Events n (%)	Survival Rate (%)	NNH/ clinical interpretation
**Leisure Time Physical Activity Pattern**					
Started (n = 53)	1.00 (reference)		4 (7.5%)	92,5	–
Sedentary (n = 48)	3.69 (1.14–11.93)	0.029	11 (22.9%)	77,1	6
Quit (n = 34)	4.61 (1.47–14.49)	0.009	12 (35.3%)	64,7	3
Active (n = 141)	2.34(0.80–6.86)	0.121	22 (15.6%)	84,4	12
**Demographic Characteristics**					
Sex (male)	1.03 (0.54–1.97)	0.922	—		No significant difference
Age (years)	1.16 (1.10–1.23)	<0.001	—		Increased age associated with higher risk
**Education**					
High	1				No significant difference
Low	0.87 (0.39-1.93)	0.723			No significant difference
Medium	0.46 (0.21-1.06)	0.070			No significant difference
**Cardiovascular Risk Factors**					
BMI	0.97 (0.91–1.04)	0.394	—		No significant association
Hypertension	1.59 (0.63–3.99)	0.326	—		No significant association
Diabetes	1.71 (0.91–3.20)	0.093	—		No significant association
Smoking (pack-years)	0.99 (0.97–1.01)	0.205	—		No significant association
Alcohol (g/week)	1.00 (0.998–1.005)	0.439	—		No significant association

## Discussion

In summary, the study found that diabetes was most frequently developed by the “sedentary” study subjects and “office worker” groups in general during P1, while it seemed that fewer cases were observed among “occupationally physically active” subjects. Hypertension incidence was lowest among “office workers” but not among “occupationally physically active” in the “active” group during P1 follow-up. Non-fatal CVD events were more common among the “quit” group and specifically among “occupationally physically active” in the OPA groups during P1 follow-up. During P2 follow-up CVD mortality was increased in the “sedentary” group compared with the “active” group. This was also evident among “office workers”, where CVD mortality was increased in both the “sedentary” and the “quit” groups. Total mortality increased during P2 for “office workers” in the “sedentary” or “quit” groups compared to the “started” group.

### Morbidities

The role of physical activity in reducing the risk of type 2 diabetes is well-documented. Our findings align with previous research. [20, 21] In our study, those who continued to exercise had the lowest incidence of diabetes, while sedentary individuals had the highest. This trend was observed in both “office workers” and those with physically active jobs. Notably, fewer new diabetes cases were found among “occupationally physically active” individuals, especially those who changed their leisure-time physical activity (LTPA) habits (“started” or “quit”). This is also supported by the meta-analysis of Aune et al., which found that all types of physical activity were associated with a reduced risk of diabetes. [21] Also, a recent study reported that low OPA was associated with an increased risk of type 2 diabetes, particularly among overweight and obese individuals. [22] In our study, occupational physical activity likewise appeared to be associated with a lower incidence of diabetes compared with “office workers”.

Participants who were regularly active during leisure time had the lowest incidence of HTN. At follow-up, “active” office workers had the lowest incidence of HTN, but this was not seen in the “active” group of occupationally physically active individuals during P1. Previous meta-analyses have shown a dose-dependent relationship between physical activity and reduced hypertension risk. [23, 24] Our findings are consistent with this, as an active lifestyle during leisure time was protective against HTN in the whole population and among “office workers”. A recent study found that moderate OPA decreased HTN risk, while high OPA increased it. [25] In our study no significant differences in HTN were observed among occupationally physically active subjects. This may be due to fact that we could not distinguish the intensity of occupational physical activity. Only some subjects were in physically demanding occupation, while the most were in moderately physically active occupation. As a result, the positive effects of moderate OPA on hypertension and the negative effects of heavy OPA could be confounding the results.

Over a 20-year follow-up (P1), we found that regular, moderate exercise significantly reduced the incidence of MetS, regardless of occupational background. A 2011 study found that sedentary time was associated with MetS independently of physical activity. [26] A previous meta-analysis with the longest follow-up, over 10 years, found that high and moderate LTPA was associated with reduced risk of MetS. The study noted that a high amount of LTPA decreased the risk of MetS regardless of intensity, time or sex. [27] These findings are consistent with ours.

### Non-fatal and fatal CVD events and total mortality

Previous research has shown that overall physical activity reduces the risk of cardiovascular disease in a dose-dependent manner. [28, 29] A previous study found that meeting the 2008 physical activity guidelines for Americans reduces fatal cardiovascular disease (CVD) events. [30] Furthermore, increasing physical activity from any baseline level over a five-year period has been associated with a lower risk of CVD and all-cause mortality during a 10-year follow-up. [11] Additionally, individuals who remain or become inactive have a higher risk of fatal CVD events compared with those who are physically active, whereas the risk does not differ between those who initiate and those who maintain regular exercise. [12] Other studies have also shown that increasing or maintaining LTPA lowers the risk of heart attack and heart failure and predicts lower overall and cardiovascular disease (CVD) mortality compared with reducing LTPA or remaining sedentary. [9, 10] Gao et al. further observed that sedentary behavior at work is associated with higher overall mortality, and that alternating sitting and non-sitting time at work or increasing LTPA reduces mortality risk. [9] A meta-analysis reported that high physical activity (60–75 minutes/day) during leisure time could eliminate the mortality risk associated with high total sitting time. [13] More recently, an accelerometer-based study reported that varying amounts and intensities of physical activity can mitigate the mortality risk associated with prolonged sedentary behavior, and that high sedentary time increases CVD mortality only among individuals with low levels of high-intensity LTPA. [31] We observed a similar finding in our study. Non-fatal CVD events during P1 follow-up were more common among those who stopped exercising compared to others. Stopping leisure time physical activity in middle age was associated with a higher risk of non-fatal CVD events, especially among those who were occupationally physically active. Nevertheless, CVD mortality was higher in the “sedentary” group compared with the “active” group in the overall study population. This association was particularly evident among office workers, where both the “sedentary” and “quit” groups were associated with higher CVD mortality when occupational physical activity (OPA) groups were analyzed separately. In contrast, among “occupationally physically active” individuals, no differences between groups were observed, and survival rates were more favorable across all groups. The differences remained statistically significant even after adjustment for education. Maintaining active LTPA was associated with reduced total mortality compared with being “sedentary”, particularly among “office workers”, who were more sedentary also during working hours. Moreover, among “office workers,” those who “started” regular LTPA during the P1 follow-up were associated with lower mortality in the subsequent years (P2) compared with those who “quit” regular LTPA or remained “sedentary.” These findings could not be totally explained by differences in non-fatal CVD events or comorbidities during the P1 follow-up, as no statistically significant differences were observed between groups, although the number of non-fatal events appeared to be higher even among occupationally physically active participants. Our results are consistent with previous studies and emphasize the importance of maintaining or initiating regular physical activity, especially for those engaged in sedentary occupations [9, 13, 32, 33].

While the benefits of LTPA are well established, those of OPA are less understood. Recent meta-analyses suggest that only non-occupational PA is associated with a lower risk of cardiovascular disease and total mortality. [32, 34] Higher OPA is linked to an increased risk of cardiovascular disease and total mortality in other studies, especially in males. [1, 4, 32, 35–37] Previous meta-analyses have also noticed that LTPA was beneficial at all OPA levels but smaller and less certain with higher levels of OPA. [5, 33] In our previous study, we also found that high LTPA could even be harmful for those in physically demanding occupations. [6] In our current study, “occupationally physically active” subjects did not have increased total or CVD mortality in the following years, regardless of changes in their leisure-time physical activity (LTPA) habits, and despite an increase in non-fatal CVD events, partiularly in the “quit” group. In contrast, “office workers” who reduced physical activity (“quit”) or remained inactive (“sedentary”) had an increased risk of CVD mortality, and among the “sedentary”, the risk of all-cause mortality was also elevated. This may support the importance of maintaining or initiating regular physical activity in both primary and secondary prevention. It may also suggest that previous moderate OPA, by increasing the overall level of physical activity, could even be beneficial for health. This is because participants classified as “occupationally physically active” appeared to have fewer adverse events in terms of CVD and all-cause mortality compared with those classified as “office workers”. Good secondary prevention does not fully explain why total mortality or fatal CVD events are not increased in occupationally active subjects who had the most CVD events during P1. Consistent with our findings, Dalene et al. reported that, after adjusting for socioeconomic status and other confounders, both high and moderate levels of OPA were associated with lower risks of CVD and overall mortality. [4] Moreover, a recent individual participant data (IPD) meta-analysis showed that LTPA was associated with reduced all-cause and cardiovascular mortality across all OPA groups, although the protective effect appeared less certain at higher OPA levels, highlighting particularly clear benefits among those sedentary at work. [33] In line with this, Gao et al. demonstrated that less occupational sitting time was independently linked to lower mortality rates. [9]

### Strength and limitations

The main strength of this study is its long follow-up period. Numerous detailed examinations and measurements allowed us to consider confounding factors broadly. However, we encountered also some limitations.

Self-reported physical activity is subject to both random and systematic misclassification. Recall inaccuracies and social desirability bias may lead participants to overestimate or underestimate their actual activity levels. A key limitation in the assessment of occupational physical activity (OPA) is the absence of follow-up data at 20 years. As most participants were retired by the follow-up, their occupational status was no longer applicable. Thus, the baseline status represented the lifelong burden of OPA. This limited our ability to estimate OPA exposure duration and likely introduced non-differential misclassification. Unequal retirement timing across groups may also have biased interaction analyses between OPA and LTPA. Although leisure-time physical activity (LTPA) was measured at both baseline and follow-up, the 20-year gap between assessments leaves potential variation in activity levels unaccounted for. This may have led to exposure misclassification, especially for participants whose activity fluctuated over time, thereby weakening associations with health outcomes.

In this 20-year follow-up, only 599 of the original 1,045 participants attended the follow-up visit, which could introduce a risk of misclassification due to selective attrition. However, at the follow-up 813 were alive and residing in Finland at the end of the follow-up period. Thus, the overall participation rate was 73.7%. Those who participated were generally younger and healthier and this might suggest a healthy user bias. Furthermore, the high baseline prevalence of cardiovascular disease in the “Quit” group could indicate reverse causation, whereby deteriorating health may have led to cessation of physical activity rather than resulted from it. Additionally, we were unable to account for baseline physical performance, which may be prognostically relevant and associated with physical activity behavior and long-term health outcomes.

We conducted sensitivity analyses by applying different thresholds for physical activity (PA). For occupational physical activity (OPA), we first categorized participants into three groups (no OPA, moderate OPA, and high OPA). The overall results did not materially change across these categories. Findings in the moderate OPA group were comparable to the main results, whereas the small sample size in the high OPA group limited reliability and interpretability. Therefore, combining OPA categories was considered justified. Although this approach did not allow us to examine the paradox of physical activity. For leisure-time physical activity (LTPA), participants were initially divided into three categories (no exercise, light regular, and active regular). We then constructed nine change groups reflecting increases, maintenance, or decreases in activity across these categories. Again, this reclassification did not substantially alter the main results. Thus, combining LTPA groups was considered justified to simplify the study design. Overall, leisure-time physical activity among study subjects was relatively low, and the amount of exercise in the active group remained significantly lower than WHO and PAG recommendations [7, 8].

## Conclusion

Maintaining or initiating a LTPA seems to be beneficial for both office workers and those who are physically active at work. Particularly among individuals with low OPA at work, maintaining low LTPA or reducing LTPA was associated with an increased risk of CVD and all-cause mortality during long-term follow-up. In contrast, among those with moderate to high OPA at work, reducing LTPA was associated with an increased risk of non-fatal CVD events. Consistently engaging in physical activity throughout middle age was associated with lower overall mortality and promoting long-term health.
